# The Impact of Muslim University Students’ Religious Beliefs on Willingness to Access Mental Health Care: A Qualitative Study

**DOI:** 10.1192/bjo.2026.11124

**Published:** 2026-06-30

**Authors:** Mohammed Faisal Shahin, Kathleen Kendall, Shmma Quraishe, Mohammed Ahmed, Seherish Abrar

**Affiliations:** 1 University of Southampton, Southampton, United Kingdom; 2 Woolf Institute, Cambridge, United Kingdom; 3 St Edmund’s College, University of Cambridge, Cambridge, United Kingdom; 4 University of Cambridge, Cambridge, United Kingdom

## Abstract

**Aims:**

Muslims in the United Kingdom face significant barriers to accessing mental health care. Cultural and religious practices influence how Muslims access mental health care and interpret causes of mental illnesses. However, current literature does not clearly separate cultural and religious influences on help-seeking, making it difficult to determine how each factor independently influences help-seeking behaviours.

The overarching aim of this study was to explore how Islamic beliefs influence mental health help-seeking for Muslim university students.

**Methods:**

Participants were recruited through convenience, purposive and snowball sampling. Semi-structured interviews discussing mental health and Islam were conducted with participants who identified as Muslims living in the UK. Reflexive inductive thematic analysis was used to analyse interview transcripts.

**Results:**

Nine Muslim university students, eight of whom studied medicine, were recruited. Five major themes were identified:

- Generational differences in understanding mental illness.
- Relationship between culture and religion.
- Mental health and the supernatural.
- Choosing a mental health service.
- Gender differences.

A key finding was the existence of a ‘dual lens’; participants demonstrated high mental health literacy whilst simultaneously holding beliefs in supernatural causes of mental illness. Thisresulted in a circumstantial preference for mental health care. Whilst National Health Service (NHS) services were the first resort for mental illnesses deemed purely biomedical, they were avoided if participants believed supernatural factors were causing the illness. This was due to a fear of scepticism from clinicians and perceptions of a lack of NHS cultural competency. Instead, participants preferred to seek help from Muslim mental health organisations (MMHOs). But access to MMHOs was hindered by a lack of awareness of these services.

**Conclusion:**

Islamic beliefs can act as a strong motivator for help-seeking for the participants, though this is often overshadowed by cultural stigma. To address patients’ ‘dual lens’ framework, clinicians must adopt a ‘biopsychosocial-spiritual’ model that validates religious beliefs as part of holistic care. Increased collaboration between the NHS and MMHOs is crucial to bridge the current service gap and increase awareness in the Muslim community of the mental health support available.

